# Association between Thyroid Profile Levels and Lymph Node Metastasis in Papillary Thyroid Carcinoma: A Retrospective Study

**DOI:** 10.3390/reports7030078

**Published:** 2024-09-16

**Authors:** Yu-Shan Hsieh, Ting-Teng Yang, Chung-Huei Hsu, Yan-Yu Lin

**Affiliations:** 1 School of Nursing, National Taipei University of Nursing and Health Sciences, Taipei City 11230, Taiwan; 2 Department of Research, Taipei Medical University Hospital, Taipei City 11030, Taiwan; 3 Division of Gastroenterology and Hepatology, Department of Internal Medicine, Taipei Medical University Hospital, Taipei City 11030, Taiwan; b101101009@tmu.edu.tw; 4 Division of Endocrinology and Metabolism, Department of Internal Medicine, Taipei Medical University Hospital, Taipei City 11030, Taiwan; chhsu@tmu.edu.tw (C.-H.H.); vincent_elva@hotmail.com (Y.-Y.L.)

**Keywords:** thyroid cancer, free thyroxine, thyroid-stimulating hormone, lymph node metastasis, papillary thyroid carcinoma

## Abstract

**Background:** Thyroid cancer is the most common endocrine carcinoma, accounting for 3.26% of all cancers. The most histologically, well-differentiated thyroid cancer is papillary thyroid carcinoma (PTC). Although PTC is regarded as an indolent tumor, a portion of the cancer cells metastasize to lymph nodes around the thyroid gland. Lymph node metastasis (LNM) is a critical risk factor for tumor recurrence in PTC, which strongly affects disease prognosis and the quality of life. **Methods:** This study aims to examine how differences in the level of the thyroid profile and other risk factors may influence LNM incidence in patients with PTC in Taiwan. We carried out a single-center retrospective study. These PTC patients were retrospectively reviewed by the Department of Endocrinology from 2016 to 2019. A total of 165 patients were included in our research. **Results:** The findings revealed a close relationship with the level of free thyroxine (FT4), the level of the thyroid-stimulating hormone (TSH), and lymph node metastases. The correlation in terms of FT4 (*p* = 0.005) and TSH (*p* = 0.417) with LNM was found as a result of the univariate regression analysis. In the multiple regression analysis, the findings revealed a close relationship between LNM, FT4 (*p* < 0.001), and TSH (*p* = 0.008). **Conclusions:** Although the predictability of the TSH should be examined further, the association between LNM and FT4 or TSH should not be ignored. The results could help guide decision-making and patient counseling, using the level of serum FT4 or the TSH as a possible predictive factor of the LNM in PTC.

## 1. Introduction

According to global cancer statistics, thyroid cancer is the eleventh highest in terms of incidence, with 586,202 cases worldwide. The global incidence for women is 10.2 per 100,000 people, which is three times that of men [1]. 

In Taiwan, thyroid cancer is the most common endocrine carcinoma, accounting for 3.26% of all cancers. The most histologically, well-differentiated thyroid cancer is papillary thyroid carcinoma (PTC) [2]. In Taiwan, thyroid cancer is the most common malignancy of the endocrine glands, with its incidence increasing 4-fold over the past two decades. PTC comprises 91% of all newly diagnosed thyroid cancers in Taiwan [3]. The etiology of thyroid cancer has not been fully elucidated. Evidence suggests that excess body weight, greater height, hormonal exposure, and certain environmental pollutants may cause malignant changes, but the only well-established risk factor is radiation [4]. According to previous studies, the increased serum level of free thyroxine (FT4) or the thyroid-stimulating hormone (TSH) have been reported, which also plays an important role in the demonstration of thyroid functions and has a predictive effect in regard to malignancies of the thyroid [5]. 

Although PTC is regarded as an indolent tumor, a portion of the cancer cells metastasize to lymph nodes around the thyroid gland [5]. Lymph node metastasis (LNM) is a critical risk factor for tumor recurrence in PTC, which strongly affects the disease prognosis and quality of life [6]. Several studies have revealed that the tumor size, location, tumor extension, and the presence of microcalcifications are all independent risk factors for LNM [7,8,9,10]. However, there are few studies that further demonstrate the predictive role of T4 and the TSH in regard to LNM in PTC. To our knowledge, higher levels of the TSH (0.6–6.0 µU/mL) may be associated with a risk of papillary thyroid cancer in patients. However, could FT4 or TSH be a predictor of the LNM factor in PTC? To examine how differences in the level of the thyroid hormone may influence the incidence of LNM in patients with PTC in Taiwan, we carried out a single-center retrospective study. Based on the number of patients treated in the past, it is evident that the number of thyroid cancer patients varies greatly. This variation may be influenced by differences in the number of annual health examinations or the specific test included in health examinations. Therefore, this study adopts a retrospective approach to avoid inaccuracies in the research results that could be caused by variations in patient numbers in a single year.

## 2. Materials and Methods

The Taipei Medical University Hospital (TMUH) Institutional Review Board for Clinical Research approved this study (No. N202004067), authorizing us to study LNM findings in patients with PTC. 

### 2.1. Data Collection 

For this retrospective study, no physical examinations, specimen collection, or other interventions were required. We only evaluated patient outcomes in terms of LNM incidence. Data from January 2016 to December 2019 were collected after a reading of the medical records took place. We specifically focused on patients with PTC who had an LNM initial diagnosis (the patients who received diagnostic surgery, which involves central neck dissection for all PTC patients to diagnose lymph node metastases). The serum FT4 and the TSH of patients were collected before any medical treatment. 

**Figure 1:**
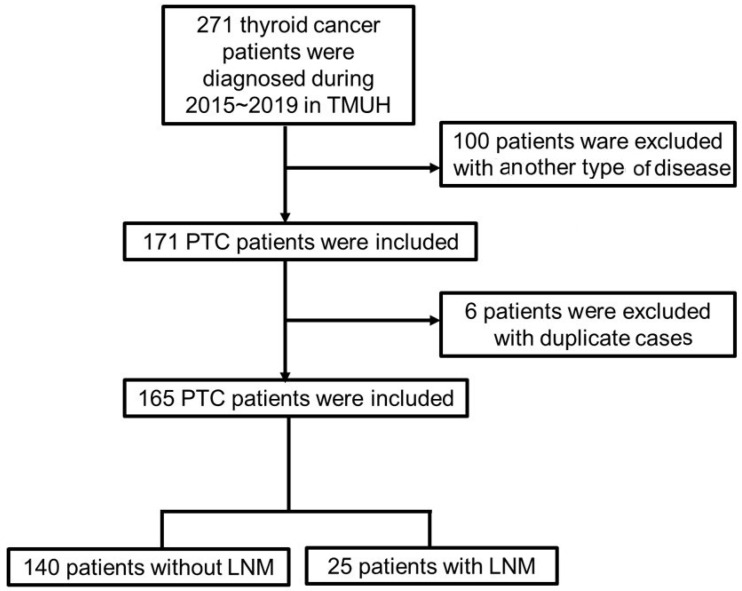
Flow diagram of data extraction. PTC: papillary carcinoma; LNM: lymph node metastasis.

Patient data meeting the following criteria were collected: (1) confirmed PTC at initial diagnosis (ICD-10 code: C73.0), (2) regular follow-up in the outpatient department, and (3) presence or absence of LNM. The exclusion criteria were: (1) the initial diagnosis was another type of thyroid carcinoma, (2) the patient has other cancers or other types of thyroid carcinoma simultaneously, and (3) the patient has received any other type of medical treatment. The flow diagram of the data extraction process is presented in Figure 1.

### 2.2. Statistical Analysis 

The data are expressed as the mean and median. The Chi-square test was used for the results that had a nominal scale. Statistical analysis was performed using IBM SPSS Statistics 22 (IBM Corp, Armonk, New York, NY, USA). Data distributions were analyzed by the Kolmogorov–Smirnov test for normality. Non-parametric statistics, which were the Mann–Whitney U test with the Kruskal–Wallis test, were used to calculate whether any group differences existed in the groups of patients, the post hoc test used was Dunn’s post hoc test. A *p*-value of <0.05 was considered significant. In the univariate regression, we defined a *p*-value of <0.5 as significant for the data selection. In the multiple regression analysis, we defined a *p*-value of <0.05 as significant.

## 3. Results

From June 2015 to December 2019, a total of 277 patients were diagnosed as having thyroid cancer at the TMUH in Taiwan. During data preparation, we excluded 100 patients who were diagnosed with medullary, follicular, and anaplastic thyroid cancer, and six patients, who were duplicate cases. Finally, 165 patients were included in our research, among whom, 140 were diagnosed as having PTC without LNM, and 25 had LNM at diagnosis or as a result of the operation (LNM was discovered postoperatively). All patients received an operation with a bilateral total thyroidectomy; LNM patients received a bilateral total thyroidectomy and lymph node dissection.

### 3.1. The Characteristics and Initial TNM Stage in Participants

The 165 patients included 159 females and 6 males. There were 137 females and 3 males in the PTC without LNM group (non-LNM group), and the mean age was 38.51 years old. There were 22 females and 3 males in the PTC with LNM group (LNM group), and the mean age was 44.08 years old, and the total *p*-value was 0.072. There was also no significant difference between the groups for each year. 

In the initial TNM stage, whether for the non-LNM (65%) or LNM group (14%), most of the participants had T1 stage (79%). In the non-LNM group, T2 and T3 stages were 9% and 10% higher than in the LNM group (1.2% and 0.6%). Whether the patients had LNM or not, the number of T1 stages was more than the other T stages (Table 1).

### 3.2. Incidence of LNM in Patients with PTC

The annual incidence rates were calculated by the following formula: the annual number of patients in the LNM group divided by the annual total number of patients multiplied by 100 (%). The incidence rates for LNM from 2015 to 2019 were as follows: 2015, 28%; 2016, 3%; 2017, 20%; 2018, 14%; and 2019, 26%. The 5-year mean incidence was 16% and the median incidence was 20% (Table 1).

### 3.3. Baseline Characteristics in the Thyroid Function Test of the Study Subjects

When comparing the chronic thyroid-related disorder history between the patient groups, 35% of non-LNM group patients had hyperthyroidism and 13% had hypothyroidism, whereas 50% of LNM group patients had hyperthyroidism and 12% had hypothyroidism. Whether the patients had LNM or not, the percentage of hyperthyroidism was higher than that of hypothyroidism (Table 1). Furthermore, the level of serum FT4 and TSH were not different between the non-LNM and LNM groups throughout the years (2015–2019). However, the FT4 level showed a significant difference between the non-LNM and LNM groups in 2015 (*p* = 0.046) and 2017 (*p* = 0.018) (Table 2).

### 3.4. Regression Analysis in Terms of Factors Affecting the Initial Tumor Size and Initial Lymph Node Metastasis

The findings revealed a close relationship between the level of FT4, the level of TSH, and lymph node metastases. However, the correlations in terms of FT4 (*p* = 0.767) and TSH (*p* = 0.771) with the tumor size were not significant. The correlations in terms of FT4 (*p* = 0.005) and TSH (*p* = 0.417) with LNM were found through univariate regression analysis (Table 3).

Furthermore, the tumor size was not found to be significant in relation to LNM in the univariate regression analysis (*p* = 0.903). In the multiple regression analysis, the findings revealed a close relationship between LNM, FT4 (*p* < 0.001), and TSH (*p* = 0.008) (Table 4).

## 4. Discussion

The presented study was a retrospective, single-institutional study. From June 2015 to December 2019, 277 patients were diagnosed with thyroid cancer at TMUH in Taiwan. The findings revealed a close relationship between the level of FT4. In the multiple regression analysis, the findings revealed a close relationship between LNM, FT4, and TSH. This indicates that in Taiwan, the TSH can indeed serve as a reference marker for predicting LNM in PTC patients.

However, 3% of the LNM rate in 2016 stands out, as presented in Table 1. It is lower than the rate for the other years. The pattern of patients receiving health examinations deviates from the norm in this year because some medical examination organizations integrate thyroid ultrasonography into routine examinations. 

Although the result in 2016 is lower than the rate for other years, we still found that the incidence of LNM in PTC patients was relatively lower in this single center in Taiwan than in China [11], South Korea [12], and Japan [13]. However, the incidence of LNM in PTC patients was relatively higher than in Italy, a European country [14].

### 4.1. The Role of the Thyroid Profile in the Incidence of LNM in Patients with PTC

Consideration of both the iodine intake and thyroid function are important to assess the PTC risk that has been reported. Thyroid function can be observed by thyroid function tests (TFTs), which include measuring the TSH (also known as thyrotropin) and FT4 (thyroxine). According to a previous study, excessive iodine intake and high serum FT4 levels may have more of a synergistic effect on PTC risk and LNM than the group with low levels [15]. 

There are possible mechanisms that could indicate high thyroid hormone levels and their role in increasing the onset or disease progression of PTC [16]. An in vitro study involving thyroid cancer cells indicates that the thyroid hormone could activate mitogen-activated protein kinase expression, leading to cancer cell proliferation. Moreover, there have been studies that suggest that higher levels of FT4 in the blood may be associated with an increased risk of developing solid cancers [17] or cystic lymphangiomas (ectopic thyroid tissue) [18]. The actions of the thyroid hormone could chemically modify gene expression in thyroid cancer cells. T4 induces radiotherapy resistance via the induction of a conformational change in the integrin in cancer cells [19]. In our results, patients also showed an abnormal level of TSH. A previous study indicated that both deficient and excessive iodine intake can increase TSH levels [20]. The TSH stimulates the change in Braf-V600E-induced tumor progression via the downregulation of p53 expression in PTC [21]. In the case of a low nutritional status in terms of iodine, type 1 iodothyronine deiodinase levels exhibit an abnormal increase in the thyroid, leading to an increase in plasma TSH and type 2 deiodinase activity in the thyroid [20]. However, an excessive iodine nutritional status may affect the pituitary gland’s type 2 deiodinase activity, leading to an increase in serum TSH [22]. The phenomenon is a bidirectional effect, which means that abnormal TSH could induce thyroid dysfunction (an abnormal increase or decrease) by interfering with homeostasis in cells.

However, some regional variations may also influence LNM incidence in PTC in Taiwan. Hence, we attempt to identify the most salient factors in terms of regional variation, namely dietary patterns, healthcare systems, and medication adherence, through a literature review.

### 4.2. The Role of Dietary Patterns in the Incidence of LNM in Patients with PTC

Another contributing factor to thyroid cancer rates is diet. Variations in dietary patterns may be influenced by different regional, ethnic, and lifestyle factors. In northeast Asian countries, such as South Korea and Japan, dietary iodine intake was generally higher than in other countries, because of the high seafood and seaweed consumption. In the Middle East, where iodine deficiency disorders remain a serious public health problem [23], a higher incidence of LNM in PTC was observed, compared with countries in northeast Asia. 

Taiwan is an iodine-deficient area and a supplementally salt iodization policy was implemented from 1971 to 2002. However, a study in Taiwan revealed a high prevalence (73%) [24] of the BRAFV600E gene mutation in patients with PTC, which was similar to that reported in an iodine-replete area in South Korea (73.7%) [25]. Iodine nutritional intake could influence various thyroid diseases; however, the association between iodine nutritional intake and PTC remains controversial. Among the iodine-metabolizing genes, the oncogene BRAFV600E mutation is the most prevalent, caused by a thymine-to-adenine transversion. It is the major oncogenic genetic alteration-specific target for PTC. The iodine nutritional status is involved in the occurrence of the BRAFV600E mutation and tumorigenesis.

Another recent study [26] indicated that the intake of nitrate-contaminated groundwater may increase the risk of thyroid cancer. In some rural regions of China, groundwater use remains prevalent [27]. Therefore, lifestyle factors may contribute to the higher incidence of LNM in PTC in certain regions. Although we initially believed that ethnicity may influence dietary patterns, some studies have indicated otherwise. Differences in the incidence of LNM in PTC still exist between China and Taiwan, where differences in ethnicity are minimal. In contrast, Italy and Taiwan, where the differences in terms of race are substantial, exhibited a similar incidence of LNM in PTC. 

### 4.3. The Role of Healthcare Systems in the Incidence of LNM in Patients with PTC

Apart from dietary patterns, healthcare systems may also be a key factor. We were unable to examine the broad range of lifestyle factors that may contribute to differences in dietary patterns, but differences in medication adherence should be considered. Individuals with neoplasms often require long-term treatment and may have limited access to the required medication because of the cost. As novel, more appropriate, or more expensive medications continue to emerge, their prices are likely to continue to increase. In the United States, a large proportion of the population incurs healthcare expenses beyond their ability to pay; they are, therefore, at risk of deferring medications to lower the financial burden [28]. However, the absence of significant differences in medication adherence between ethnicities is notable [29]. Education, likely a marker of health literacy, was not independently associated with medication adherence. These results highlight that the removal of financial barriers to accessing medications, particularly among vulnerable patient groups, may improve adherence to essential therapy, thus reducing morbidity and mortality [30]. Therefore, the presence of a functional and reliable healthcare system may have a crucial role in lowering the incidence of LNM in PTC.

### 4.4. The Role of Medication Adherence in the Incidence of LNM in Patients with PTC

Medication adherence in the management of diseases should also be considered. Poor adherence is associated with high healthcare costs, a lower quality of life, and less favorable health outcomes. The previous results indicated that patients with poor compliance-related TSH levels were more significantly abnormal than those displaying good compliance [31]. Motivating patients to follow treatment regimens and visit the hospital on time remains crucial in the clinical setting.

### 4.5. Limitations

Our study has some limitations. First, the number of cases (165 patients with PTC) included in this study was relatively small, which may have led to sampling bias. After 2019, the world entered the COVID-19 pandemic period, which significantly affected patients’ willingness to visit hospitals for treatment or attend regular follow-ups. As a result, this study only retrospectively analyzed medical records from 2016 to 2019. This limitation contributed to the relatively low number of patients and the shorter retrospective period. We will address this issue in further research.

Second, approximately 95% of the patients in our study were women. Thyroid cancer is more common in women than in men; hence, the skewed gender data may be an aspect that limits the generalizability of the results. Third, in order to approach real-world circumstances, the patients who had subclinical diseases were not excluded from this study. If the patients presented clinical or subclinical hyperthyroidism, respectively, their FT4 values should be in the normal range and may influence the statistics. Forth, another limitation is that we had little data on other environmental factors, such as iodine intake, stress, negative life events, and infections. Finally, the study lacked a physical examination, details on the presence of a goiter, and the recording of hypothyroidism-related clinical signs, aspects that need to be considered and documented in future studies. 

## 5. Conclusions

In conclusion, this retrospective study provides further evidence indicating that FT4 and TSH might affect LNM incidence in PTC. In clinical practice, thyroid cancer is often overlooked due to its high survival rate and low mortality. Patients may not undergo further imaging to track lymph node metastasis due to a lack of symptoms, and clinicians may only rely on blood tests to assess patient conditions. Therefore, the results of this study suggest a blood marker that can be used to predict possible lymph node metastasis in patients. This marker can be used as a reference for tracking metastasis in the future. We also describe some possible related factors, which include the healthcare system, dietary patterns, and medication adherence. A further study examining the specific criteria for the ranking of the importance of variables in the population may be necessary. Although the predictability of the TSH should be further examined, the association between LNM and FT4 or TSH should not be ignored. The results could help guide decision-making and patient counseling, using the level of serum FT4 or TSH as a possible predictive factor. 

## Figures and Tables

**Table 1:** Baseline characteristics of the study subjects.

				2015	2016	2017	2018	2019	Total
Gender (N, female)									
	Non-LNM			23 (23)	58 (58)	28 (28)	18 (17)	12 (10)	140 (137)
	LNM			8 (8)	3 (3)	7 (7)	3 (2)	5 (3)	25 (22)
Age [mean (median)]									
	Non-LNM			36.04 (36.0)	36.12 (38.0)	46.42 (39.35)	46.39 (41.91)	35.60 (42.23)	38.51 (2.33)
	LNM			47.86 (41.81)	27.00 (36.68)	29.00 (28.07)	38.67 (35.91)	45.40 (44.45)	44.08 (9.07)
	Total mean			34.68	36.13	38.12	43.75	31.5	36.84 (3.67)
	*p*-value			0.082	0.190	0.730	0.308	0.664	0.072
Chronic disease history [N, (% each group)]									
	Hyperthyroidism								
			Non-LNM	16	16	7	6	4	49 (35)
			LNM	3	2	2	1	2	13 (50)
			Total	19	18	9	7	6	61
	Hypothyroidism								
			Non-LNM	2	10	5	2	3	22 (13)
			LNM	3	0	4	0	0	3 (12)
			Total	5	10	9	2	4	25
Initial diagnosis [N (% total)]									
	Non-LNM								
		T	1	20 (71)	44 (79)	19 (68)	17 (81)	8 (50)	108 (65)
			2	3 (10)	6 (10)	2 (7)	0 (0)	4 (24)	15 (9)
			3	0 (0)	9 (15)	7 (25)	1 (5)	0 (0)	17 (10)
			4	0 (0)	0 (0)	0 (0)	0 (0)	0 (0)	0 (0)
			x	0 (0)	0 (0)	0 (0)	0 (0)	0 (0)	0 (0)
	LNM								
		T	1	8 (28)	1 (2)	7 (20)	3 (14)	4 (14)	23 (14)
			2	0 (0)	1 (2)	0 (0)	0 (0)	1 (12)	2 (1.2)
			3	0 (0)	1 (2)	0 (0)	0 (0)	0 (0)	1 (0.6)
			4	0 (0)	0 (0)	0 (0)	0 (0)	0 (0)	0 (0)
			x	0 (0)	0 (0)	0 (0)	0 (0)	0 (0)	0 (0)
LNM incidence (%)				28	6	20	14	26	16

LNM: lymph node metastasis.

**Table 2:** Baseline characteristics in thyroid function test of the study subjects. FT4: free thyroxine. TSH: the level of thyroid-stimulating hormone.

		Reference Range	2015	2016	2017	2018	2019	Total
FT4 [mean ng/dL (median)]		0.8~1.8 ng/dL						
	Non-LNM		0.7 (0.78)	1.24 (0.82)	1.27 (1.4)	1.16 (1.53)	1.47 (0.54)	1.16 (0.72)
	LNM		1.67 (1)	1.11 (1.42)	1.39 (1.88)	0.96 (1.4)	1.27 (0.83)	1.37 (0.92)
	Total		0.69 (0.73)	1.24 (1.68)	1.19 (1.63)	1.17 (1.5)	1.31 (0.61)	1.12 (0.08)
	*p*-value		0.046 *	0.292	0.018 *	0.188	0.583	0.906
TSH [mean uIU/mL (median)]		0.4~5 mIU/L						
	Non-LNM		18.99 (24.84)	30.57 (30.68)	9.56 (9.86)	14.99 (18.58)	13.13 (20.54)	15.90 (23.39)
	LNM		27.46 (24.8)	14.6 (13.75)	5.37 (9.16)	7.30 (12.83)	4.82 (7.08)	13.12 21.03)
	Total		27.01 (24.80)	14.82 (18.29)	11.77 (11.08)	7.08 (7.15)	9.66 (16.85)	14.07 (4.05)
	*p*-value		0.315	0.099	0.726	0.089	0.478	0.658

*: *p*-value < 0.05.

**Table 3:** (a) Univariate regression analysis in terms of the factors affecting the initial tumor size (T). (b) Univariate regression analysis in terms of the factors affecting initial lymph node metastasis (N).

**(a)**		
**Independent Variables**	***R* ^2^**	***p*-value**
FT4	0.005	0.767
TSH	0.052	0.771
**(b)**		
**Independent Variables**	***R* ^2^**	***p*-value**
FT4	0.480	0.005 *
TSH	0.004	0.417 *

FT4: free thyroxine. TSH: the level of thyroid-stimulating hormone. *: *p* < 0.5.

**Table 4:** Factors affecting initial lymph node metastasis (N): multiple regression analysis. FT4: free thyroxine. TSH: the level of thyroid-stimulating hormone. **: *p* < 0.01, ***: *p* < 0.001.

Independent Variables	Coefficient	Std. Error	*r*_partial_	*p*-Value
FT4	0.152	0.055	0.2191	<0.001 ***
TSH	0.003	0.002	0.1437	0.008 **

** *p* < 0.01, *** *p* < 0.001.

## Data Availability

The data used and/or analyzed during the current study are available from the corresponding author upon reasonable request. The data are not publicly available due to privacy.

## Abbreviations

Free thyroxine (FT4); thyroid-stimulating hormone (TSH); lymph node metastasis (LNM); papillary thyroid carcinoma (PTC).
